# Ageing, health and care

**Published:** 2011-10-07

**Authors:** K. Viktoria Stein

**Affiliations:** Institute of Social Medicine, Center for Public Health, Medical University of Vienna, E-mail: katharina.v.stein@meduniwien.ac.at

This book sets out to give an insight into the challenges of care for the elderly and is authored by Christina R. Victor, Director of the Healthy Ageing Programme of the Brunel Institute for Ageing Studies, at Brunel University. In seven chapters, all aspects of the topic—from epidemiology, data collection, to social and long-term care issues as well as future perspectives and trends—are introduced. The focus is clearly on the health care system of the UK, with references to international data and studies, where available. Each chapter opens and closes with key points being highlighted and activities throughout the chapters invite the reader to test their own knowledge and understanding of the topics discussed. The following paragraphs will give a summary of all the chapters.

Chapter 1 gives a concise introduction into the key concepts of health and ageing, defining health, disease, disability and impairment. The demographic key terms are also explained. The author discusses the notion of old age being named as a ‘disease’ in our society and the general misperception that old age/growing old equals disease and disability. The readers are introduced to the different viewpoints of the medical and sociological concepts on ageing and the stigmatization that goes along with the process. The broad viewpoint that is taken throughout the book, by looking at ageing from a medical, sociological, ethnic and gender perspectives, is already presented in this introductory chapter.

Chapter 2 describes the different possibilities of measuring health and mortality, as opposed to morbidity. The subject of health inequalities is introduced and the difficulties in gathering meaningful and realistic data are discussed. Even so, health inequalities diminish in older age but still influence the ageing process significantly. The author criticizes that many reports generalize older people under the age group of 65+ without taking into account further subgroups. The chapter continues with an overview of major health problems related to and associated with morbidity and mortality in older people.

Chapter 3 underscores the importance of self-evaluated health status and how older people understand health as either' an absence of illness', ‘functionality’ or ‘physical activity/fitness’. Here, the instrument of activities of daily living (ADL) are introduced and UK data presented, as well as data on the debilitating effect of chronic diseases on said activities of daily living. These issues are also discussed under the aspects of gender, ethnicity and socio-economic status and a lack of comprehensive data is identified. Even though little age-related data are available on these determinants of health, they are suggestive of significant influences of these social constructs on the health and well-being in older age. The chapter concludes with a discussion of the “4 giants of geriatric medicine: immobility, instability, incontinence, and intellectual impairment” [1].

Chapter 4 takes a closer look at the mental health and psychological wellbeing in older age. The importance of the reference framework and expectations of health in older age are highlighted, along with self-reported data on health status in old age. The data reveal that good health is very much a function of being able to live and cope independently. They also demonstrate that individual health status is often rated quite high since the expectations on health in older age are generally low, and in comparison the individual health is considered to be better than expected.

Furthermore, Victor gives an overview of data on dementia, depression, suicide, and anxiety in old age, highlighting the demographic and social changes and trends. Also discussed are the effects that mental health and disease have on the functionality in old age.

Chapter 5 introduces the under-estimated importance of older people as consumers, specifically concerning health-related lifestyles and consumption goods. A critical position is taken towards the anti-ageing medicine (market) and the social consequences this entails. Far from promoting healthy ageing, anti-ageing medicine and its associated industries sell the dream of eternal youth. The main argument against it is its lack of evidence-based medicine. Lifestyle choices concerning diet, physical activity, alcohol and smoking habits are briefly discussed, as are the questions on the existence of informed consumers and a health care market in the UK.

Chapter 6 deals with the question of care provision, explaining the different settings and localities of care and the role of the informal care givers. Victor looks closer at how needs for care are assessed, using ADL and IADL, who the users are and what kind of care is provided. The author explains the use of medical care, primarily in the form of hospital stays, versus social care and the shift that has happened towards the latter as a result of the ageing of the population and the rise of chronic disease. This shift has caused a further decline in interest of the medical field in older people and their specific health needs, since they are considered to need foremost care services, provided outside of the hospital and in the community.

The final chapter discusses the future development of health, mortality, and morbidity under the compression and expansion models and the ‘rectangularisation of mortality’. As mortality rates in early childhood and adult life decreased significantly in the developed countries, mortality occurs primarily at an advanced age. However, with chronic diseases rising and medical service provision improving continuously the question remains whether the increase in life expectancy goes hand in hand with a prolonged morbidity and whether this will reverse the trends and lead to a rise of mortality again.

Overall, the book gives an introduction into the most important issues concerning health and ageing, the evaluation and measurement as well as its social implications. Where possible, it discusses the topics under aspects of gender, socio-economic status, and ethnicity as well, always reminding the reader that too little is known about these determinants of ageing. However, the book seldom explores the raised issues in more detail and fails to pinpoint why geriatric medicine and gerontology are specialities in their own right. Controversial questions, for example on older people as consumers, anti-ageing or the conflict between health and social care showcase the many unresolved and highly disputed issues around healthy ageing, however, the author mostly scratches the surface and leaves the reader with a feeling of missed potential by not going into more detail. While the book does give a comprehensive overview of the key questions concerning healthy ageing, it fails to shed any new light or introduce new aspects on the topic. Problems of integration of services or transition between sectors are not considered at all. Hence, this book is ideal for professionals and academics who have not previously worked on the subject and want to get a first introduction. One of its strongest points is the further reading suggestions and its literature references which list all the seminal publications in the field. It also introduces open research questions and raises many important issues, even if it fails to discuss them adequately.
